# Supplementary material for: Crystal structure of progeria mutant S143F lamin A/C reveals increased hydrophobicity driving nuclear deformation
Source: Commun Biol. 2022 Mar 25;5:267. doi: 10.1038/s42003-022-03212-3 (PMC8956589; doi:10.1038/s42003-022-03212-3)
Supplement: Supplementary file 2 — Supplementary information [file 42003_2022_3212_MOESM2_ESM.pdf]

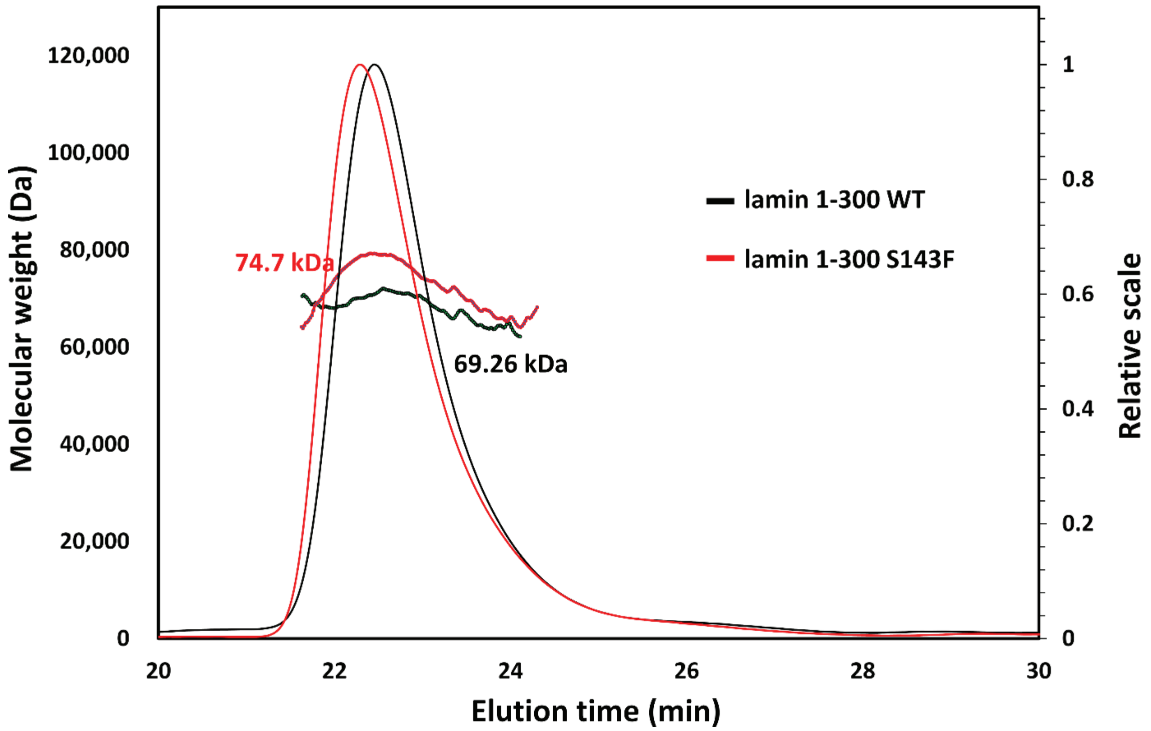

Supplementary Figure 1. Analytical size exclusion chromatography coupled with multi-angle light scattering (SEC-MALS) profiles of the wild-type (black) and S143F mutant (red) proteins of the lamin 300 fragment. The protein samples (wild-type and S143F mutant protein; 2 mg/ml) were applied to a Superdex 200 Increase 10/300 GL column (GE Healthcare) in 20 mM Tris (pH 7.5) buffer containing 150 mM NaCl.

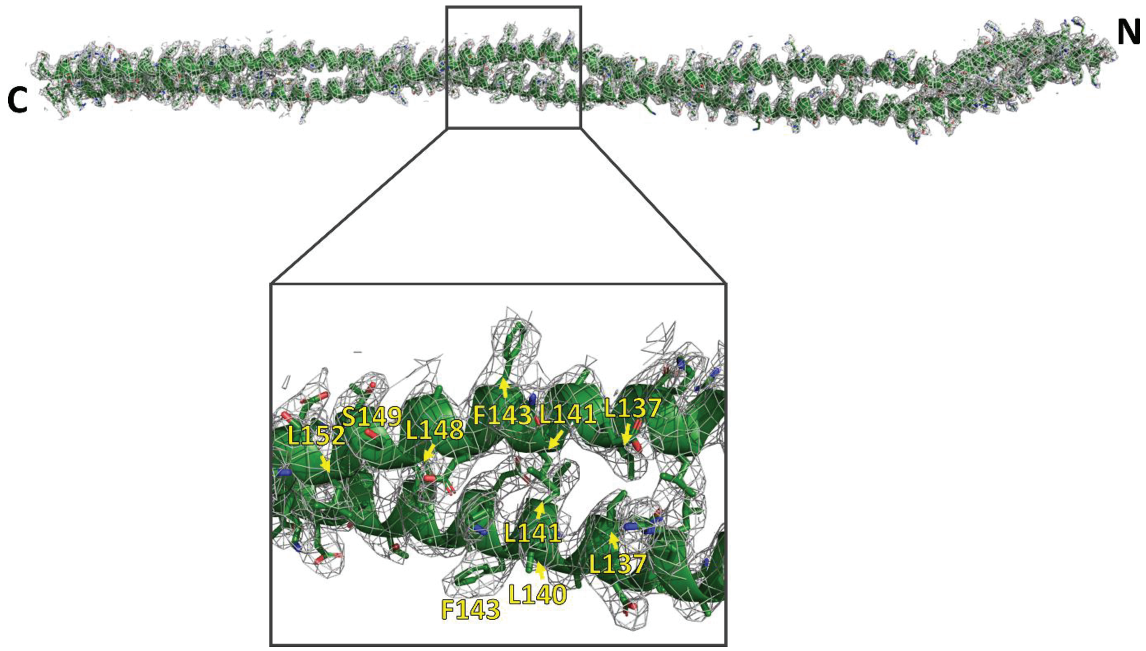

Supplementary Figure 2. Observed electron density map of lamin S143F crystal structure. The lamin S143F molecules are represented with the electron density (gray). The hydrophobic residues on the contact region for the X-shaped interaction are labelled.

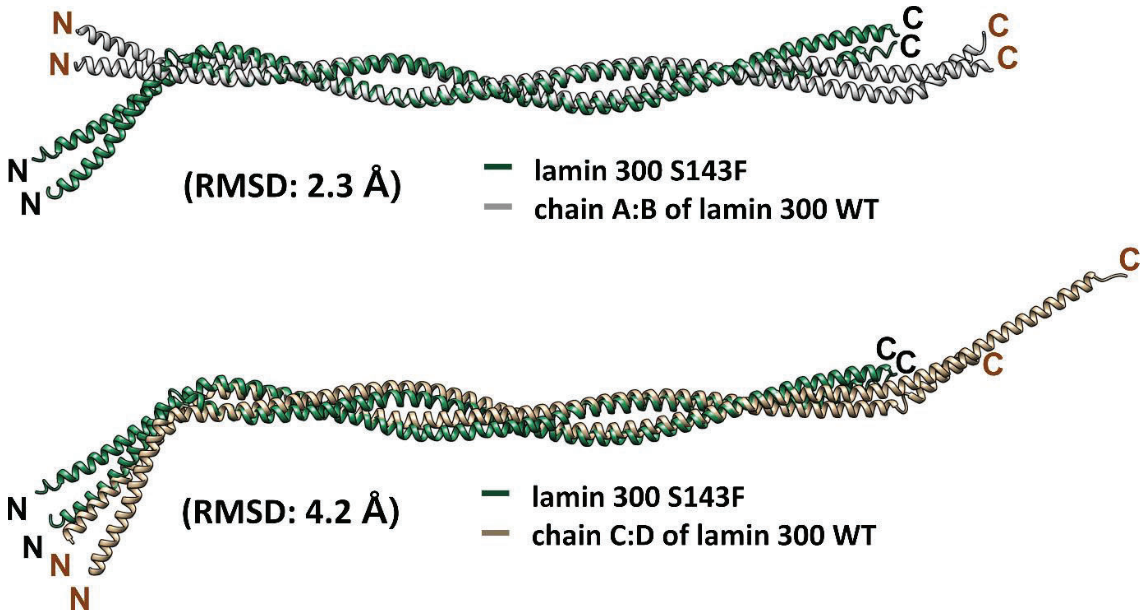

Supplementary Figure 3. Structural superposition between the coiled-coil dimers of the wild-type (PDB ID: 6JLB, chain A:B; grey, chain C:D; wheat) and the S143F mutant lamin 300 fragments (this study; green).

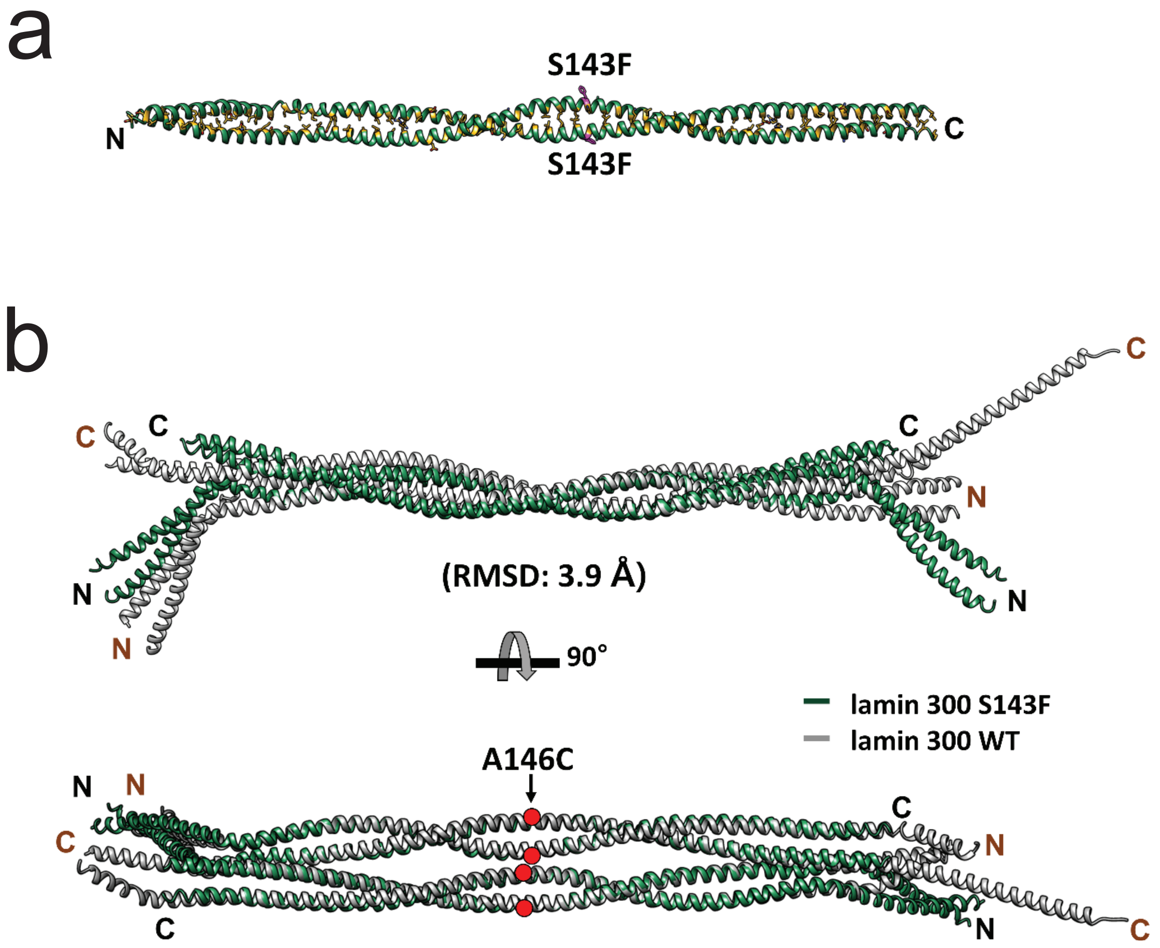

Supplementary Figure 4. The structures of the wild-type and S143F mutant lamin 300 fragments. (a) The inter-helical hydrophobic residues at the *a* and *d* positions of the heptad repeat are shown as yellow stick representations. The Phe143 residues at the *c* position of the heptad repeat are in the magenta stick representations. (b) Structural superposition between the A11 tetramers of the wild-type (PDB ID: 6JLB; grey) and S143F (PDB ID: 7D9N; green) lamin 300 fragments. The Ala146 residues are highlighted in a red circle.

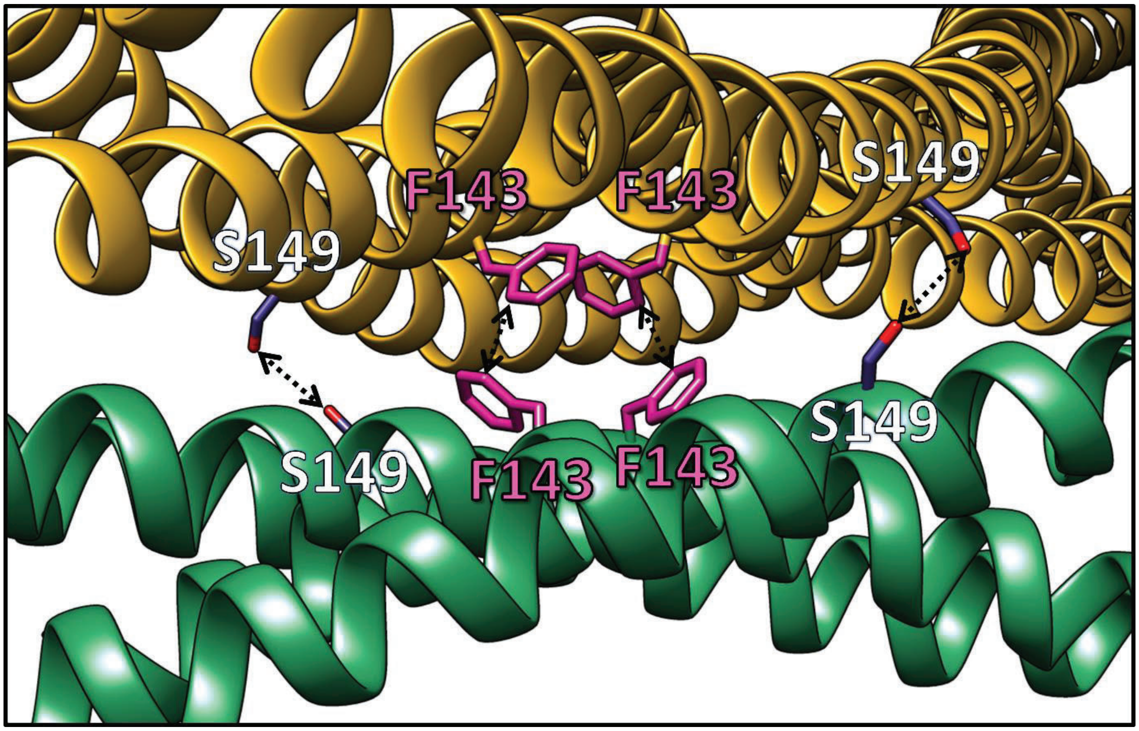

Supplementary Figure 5. The positions of Ser149 and Phe143 in the X-shaped interaction between the two A11 tetramers. The Phe143 and Ser149 residues are depicted in the magenta or violet stick representations, respectively. Dotted lines indicate the shortest distances between the Phe143 residues or Ser149 residues.

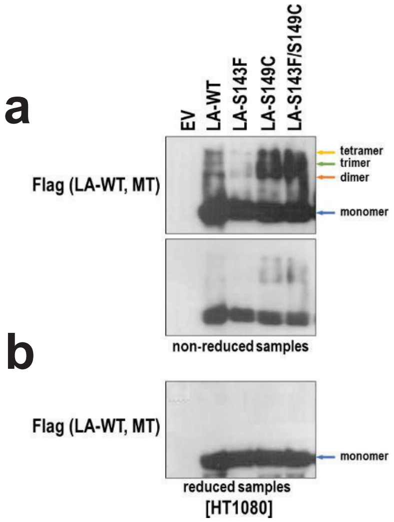

Supplementary Figure 6. The oligomeric states of the overexpressed lamin A/C proteins (LA) in HT1080 cells. (a) Western blot from SDS-PAGE gel of wild-type and mutant lamin A/C proteins (S143F, S149C, and S143F/S149C) indicates monomer and oligomer states. Oligomeric states of lamin A/C proteins are marked as different coloured arrows. The inactivated forms of wild-type and mutant lamin A/C proteins with heat-treatment and reducing agent are presented in (b).
