# Supplementary material for: Crystal structure of progeria mutant S143F lamin A/C reveals increased hydrophobicity driving nuclear deformation
Source: Commun Biol. 2022 Mar 25;5:267. doi: 10.1038/s42003-022-03212-3 (PMC8956589; doi:10.1038/s42003-022-03212-3)
Supplement: Supplementary file 3 — Description of Additional Supplementary Files [file 42003_2022_3212_MOESM3_ESM.pdf]

Description of Additional Supplementary Files

File name: Supplementary Data 1
Description: PDB structure coordinates for the S143F form of lamin A/C.

File name: Supplementary Data 2
Description: MTZ file.
